# The tactile perception of transient changes in friction

**DOI:** 10.1098/rsif.2017.0641

**Published:** 2017-12-06

**Authors:** David Gueorguiev, Eric Vezzoli, André Mouraux, Betty Lemaire-Semail, Jean-Louis Thonnard

**Affiliations:** 1 Institute of Neuroscience, Université catholique de Louvain, 1200 Brussels, Belgium; 2 Univ. Lille, Centrale Lille, Arts et Metiers ParisTech, HEI, EA 2697 - L2EP - Laboratoire d'Electrotechnique et d'Electronique de Puissance, 59000 Lille, France; 3 INRIA Lille Nord-Europe, 59650 Villeneuve d'Asq, France; 4 Cliniques Universitaires Saint-Luc, Physical and Rehabilitation Medicine Department, Université catholique de Louvain, 1200, Brussels, Belgium

**Keywords:** friction, Weber fraction, tactile displays, ultrasonic vibration, skin humidity, squeeze film

## Abstract

When we touch an object or explore a texture, frictional strains are induced by the tactile interactions with the surface of the object. Little is known about how these interactions are perceived, although it becomes crucial for the nascent industry of interactive displays with haptic feedback (e.g. smartphones and tablets) where tactile feedback based on friction modulation is particularly relevant. To investigate the human perception of frictional strains, we mounted a high-fidelity friction modulating ultrasonic device on a robotic platform performing controlled rubbing of the fingertip and asked participants to detect induced decreases of friction during a forced-choice task. The ability to perceive the changes in friction was found to follow Weber's Law of just noticeable differences, as it consistently depended on the ratio between the reduction in tangential force and the pre-stimulation tangential force. The Weber fraction was 0.11 in all conditions demonstrating a very high sensitivity to transient changes in friction. Humid fingers experienced less friction reduction than drier ones for the same intensity of ultrasonic vibration but the Weber fraction for detecting changes in friction was not influenced by the humidity of the skin.

## 1. Introduction

Frictional forces experienced when our body interacts with the environment provide essential sensory cues to adapt our behaviour. We rely daily on these sensory cues, for example when we need to feel the smoothness of the fabric before buying a clothing, or when we slide our finger against the screen of a smartphone. These frictional signals are induced by the complex contact mechanics occurring at the skin–fingertip interface during tactile interaction. The forces generated by the skin–surface contact induce surface strains and stretches, which propagate on the skin [1–3]. These frictional patterns are turned into neural signals by mechanosensitive afferents [4–6]. They are further integrated by higher-order neurons of the somatosensory system to provide meaningful feedback for major ongoing sensory and motor processes like sensing textures and materials [7–11], avoiding slippage during object manipulation [12–15] or contributing to the sensation of pleasant touch [16,17]. Touch has been found to be extremely sensitive to differences in frictional dynamics [7,18], even when these differences are tiny [19,20]. The broad sensory relevance of frictional cues has brought a great technological interest in modulating fingertip–surface friction to produce tactile displays that are able to render virtual geometric shapes and textures on flat surfaces [21–24].

Different approaches are available to produce tactile sensations on the fingertip. The approach implemented in the present study involves ultrasonic vibrations to reduce the sliding friction between the contacting finger pad and the display [25]. Two phenomena are thought to underlie this ultrasonic lubrication: the production of a thin layer of air at the skin–surface interface (known as the squeeze film effect), and the production of an intermittent contact between the finger and the surface [26,27]. As the applications for controlled friction modulation are ever-growing [28–30], the development of a high-fidelity strategy for tactile rendering is needed but faces the limitation that little is known about how the perception of frictional cues is performed by the nervous system. To implement realistic shapes and textures, it is essential to understand which components of the frictional signal are critical for tactile sensation and how to scale their intensities according to the dynamics of the interaction. Another challenge is to understand the influence of the constantly changing properties of the fingertip on the perception of frictional changes. The present study aims at quantitatively estimating the human sensitivity to transient changes in friction and identifying some of the physiological fingertip properties influencing it.

**Figure 1.**
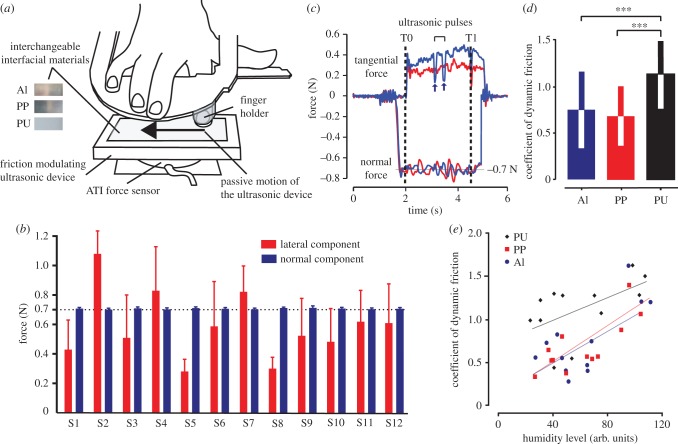
(*a*) Ultrasonic tactile display mounted on the robotic platform used to slide the surface of the display against the index fingertip. The hand and fingers are maintained by holders. The experiments were performed using three different interfacial materials: aluminium (AI), polypropylene (PP) and polyurethane (PU). (*b*) For each participant, the average tangential and normal force across all trials are plotted (mean ± s.d.). The normal force is kept constant at 0.7 N, while the tangential force shows large variations depending on the interfacial material and the mechanical properties of the fingertip. (*c*) Time-course of the transient reduction in friction induced by ultrasonic stimulation. The TF was reduced during two intervals of 100 ms occurring in the middle of the slide, which are indicated by arrows. The waveforms illustrate the force profile for a slide with and without ultrasonic stimulation, respectively. The normal force values are represented as negative for better visibility. (*d*) Coefficients of dynamic friction of the three tested materials (mean ± s.d.). (*e*) Spearman correlation between the humidity level of the fingertip and the participant's coefficient of dynamic friction for the three tested materials. (Online version in colour.)

To perform an extensive psychophysical assessment of the perception of transient changes in friction, a newly developed high-precision tactile display, capable of generating ultrasonic vibrations with controlled timing and amplitude [24], was mounted on a robotic platform designed to perform passive touch slides on the fingertip with controlled speed, position and normal force (figure 1*a*). Three different materials (aluminium, polypropylene and polyurethane) were interfaced between the fingertip and the display to test if sensitivity to frictional cues depends on their specific properties. Since bounding with water molecules has proven important for the perception of frictional cues [20], one material (aluminium) was selected hydrophilic while the other two (polypropylene and polyurethane) were hydrophobic. Moreover, two materials shared a similar stickiness (aluminium and polypropylene) while polyurethane was stickier to estimate the influence of the pre-stimulation coefficient of friction on the perception of the frictional changes. Since these differences between materials influence both the contact mechanics and the perception [18], they could induce distinct perceptual thresholds.

For a large range of stimuli and intensities that span across all modalities, the just noticeable difference, which is defined by the smallest difference in intensity that triggers a correct detection between a reference and comparison stimulus in a certain percentage of the trials, has been observed to be a constant fraction of the intensity of the stimulus. This relationship is known as Weber's Law [31]. However, examples of violation and near-misses have also been observed [32–35], especially when intensities are close to the human perceptual limits. Recent studies estimated the Weber fraction for discriminating different coefficients of dynamic friction generated on distinct surfaces to be constant, around 0.18 to 0.20 [36,37], suggesting that the ability to perceive differences in friction could follow Weber's Law. We therefore estimated the threshold at which transient frictional cues were consistently detected and compared them across three interfacial materials with different surface properties to determine what feature of the stimulus actually drives the detection, and if Weber's Law holds in the particular case of transient frictional cues. The humidity level has also been shown to influence the frictional properties of the fingertip [38,39], hence we also investigated the influence of the fingertip's moisture level on the ultrasonic reduction of friction and its perception.

## 2. Material and methods

The ethics committee on human research of Université catholique de Louvain approved the study (Virtual Prototyping of Tactile Displays, PROTOTOUCH-317100). All participants gave written informed consent. The investigation conformed to the principles of the Declaration of Helsinki and experiments were performed in accordance with relevant guidelines and regulations.

### 2.1. Participants

Data were collected from 12 healthy volunteers aged between 27 and 53 (four females). All participants performed the Edinburgh laterality test [40] and were found to be predominantly right handed with a median laterality quotient of 95 (IQR = 100–82.5). Participants were blindfolded and Gaussian white noise was played at a comfortable listening level through headsets in order to mask auditory cues. Six additional participants were recruited for a control experiment performed while the finger was kept static compared to the ultrasonic display.

### 2.2. Experimental set-up

We used a custom robotic platform designed to apply controlled stimuli to the fingertip in a passive dynamic touch condition similar to those presented in [41,42]. The robotic platform used to deliver the stimuli was based on an industrial robot (four-axis SCARA Denso HS-4535G) able to translate in three orthogonal directions. Its position is servo-controlled with a position resolution of 15 µm by a factory controller at a frequency of 1 kHz, which enables exact control of the instantaneous speed of the sliding. The subject's index finger was fixed in a support that maintains a constant angle of approximately 20° between the finger and the stimulating plate (see electronic supplementary material, movie S1). An ATI Mini 40 (ATI, USA) load cell, with a single measurement resolution of 0.01 N in Fx,Fy and 0.02 N in Fz, was mounted on the robot and served to control the normal force applied by the platform as well as to record the contact forces during stimulus presentation. The normal force is fed back by a proportional-integral-derivative (PID) controller while the tangential force would vary depending on the interfacial material and the specific properties of the fingertip. The forces, acquired at a sampling rate of 1 kHz, were averaged on minimal intervals of 100 ms and filtered bi-directionally by a fourth order low-pass Butterworth filter at 250 Hz in order to remove high-frequency noise generated by the force sensor and, thereby, improve the precision of the force measurements. The resulting sensitivity to changes in tangential force was below 0.006 N.

### 2.3. Ultrasonic friction modulation

The tangential force was modulated with a novel ultrasonic tactile display integrated with the acquisition and control chain of the robot. The display is based on a modified version of the STIMTAC [43], where the full body of the stimulator was modified to be mounted on the force sensor of the robot and the vibration amplitude of the device was controlled in closed loop. The implemented control ensures the stability of vibration amplitude with a resolution of 50 nm and a rise time of 1.5 ms. For this study, three materials were used at the skin–plate interface in separate blocks: the bare aluminium plate of the device, a glued polypropylene (PP) sheet and a glued polyurethane (PU) sheet. These materials were chosen to investigate the effect of different frictional properties of the materials on the participant's capacity to sense the modulation of the tangential force. Aluminium (hydrophilic) and PP (hydrophobic) have similar coefficients of dynamic friction (CF) but differ by their bonding of water molecules while PU has typically a higher CF and is hydrophobic.

### 2.4. General procedure

The study was made in passive dynamic touch condition in which the ultrasonic device was rubbed against the non-moving finger by a robotic platform. The robotic platform was programmed to deliver a precise stimulation of the fingertip. The stimulation consisted of a sliding of 5 cm at a speed of 2.0 cm s^−1^ with a constant normal force of 0.7 N (figure 1*b*) against the fingertip of the right index finger, which was kept still by a finger holder. Each trial consisted in the platform performing two consecutive slides against the finger. In one of the two slides, no ultrasonic vibration was induced and the participant felt the natural friction of the material. In the other slide, ultrasonic vibrations were switched on during two intervals of 100 ms, which were programmed to precisely start at 22 mm and 25 mm of sliding (figure 1*c*). The location (first or second slide) of the ultrasonic vibrations was pseudo-randomized. The ultrasonic vibrations used to induce the transient frictional transitions had peak-to-peak amplitudes of 0.1, 0.2, 0.3, 0.5 or 0.7 µm. The different intensities were repeated 10 times, in a pseudo-randomized order (total of 50 trials per session). Participants had to perform a forced-choice task to find out which of the two slides contained the two ultrasonic square modulations. We chose to deliver two ultrasonic pulses in the target slide because preliminary experiments showed a lapse rate (a constant percentage of mistakes that does not depend to the intensity of the stimulation) of more than 10% for a one pulse stimulus, probably due to the masking effect of naturally occurring frictional events and to the variability in the mechanical response of the fingertip to the ultrasonic stimulation. This lapse rate was too high to enable precise psychophysical measurements [44]. With two consecutive pulses, the lapse rate was reduced to 5%. Each participant performed the procedure for three conditions, which differed by the material placed at the tactile interface (Al, PP, PU). The order of the conditions was counterbalanced across participants.

We performed an identical experiment in static touch condition. In this control experiment, the robotic platform moved only vertically in order to establish contact between the device and fingertip, and then deliver the ultrasonic pulses to a finger that is static instead of laterally rubbed by the device. The aim of this experiment was to explore whether tactile perception could be due to an artefact related to the functioning of the ultrasonic display since there should be no sensation related to changes in friction when the finger is static against the display.

### 2.5. Computation of the force signal

The coefficient of dynamic friction (CF) was computed within the interval corresponding to 250 ms after the onset of sliding and 250 ms before the end of the sliding. The outer intervals were excluded from the analysis to avoid border effects. The CF was computed as the mean ratio between the tangential force (TF) and the normal force.

**Figure 2.**
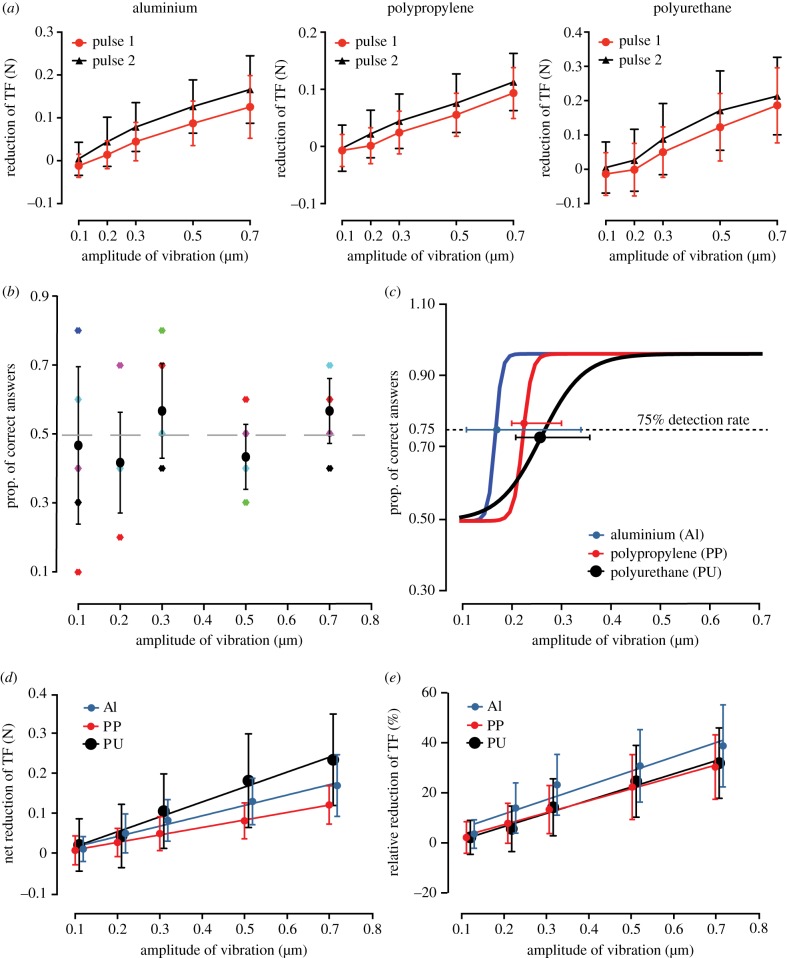
(*a*) For each trial, the two ultrasonic pulses composing the stimulation were analysed separately and the results showed that the second pulse induced a significantly higher reduction of TF (mean ± s.d.) than the first one. The same trend was observed for all three tested interfacial materials. (*b*) The stimulation and psychophysical procedure were performed on a static finger with the exact same timing and normal force as in the dynamic condition. The averaged correct answers for all the participants are plotted (mean ± s.d.) for all amplitudes as well as the individual proportions of correct answers (colour stars). (*c*) Performance at detecting the ultrasonic pulses compared to the amplitude of the ultrasonic vibrations. The psychometric function computed out of the median of the individual slopes and psychophysical thresholds (median ± IQR) was computed for each material (the three curves are slightly shifted for a better visibility: Al is accurate, PP is shifted by 0.02 upwards and PU is shifted by 0.02 downwards). (*d*) Average net reduction of TF for each material according to the amplitude of ultrasonic vibration (mean ± s.d.). A linear regression was computed for each material and plotted as a continuous line. (*e*) Average relative reduction of TF for each material (mean ± s.d.). A linear regression was computed for each material and plotted as a continuous line.

The mechanical response of the skin to the two consecutive pulses of same intensity was found to slightly fluctuate (Wilcoxon matched-pairs signed ranked test: *n* = 600, *p* < 0.0001) for all materials with the second of the two consecutive pulses triggering a larger reduction of TF in most of the trials (figure 2*a*). Thus, we used the values from the more salient of the two pulses to perform the analysis of the contact force data. For each interfacial material, the mean strength of the ultrasonic lubrication was computed by averaging the reduction of TF across all stimulations delivered to a participant. The net reduction of TF was computed by subtracting the mean TF during the 100 ms ultrasonic pulse from the mean of TF during the 100 ms pre-pulse interval. The relative reduction was defined as the percentage of reduction compared to the mean of TF during the 100 ms pre-pulse interval. It can also be expressed as a Weber fraction, which is defined by the ratio between the net reduction of TF and its initial value.

### 2.6. Humidity measurements

Each measurement of the moisture level of the fingertip was performed by averaging three repeated measures using a corneometer (CM 825, Courage + Khazaka electronic GmbH, Germany). For each experimental condition, a measure was performed before the first trial and after the last one. Since no significant difference was observed between the two repeated measures (paired *t*-test: *N* = 12, d.f. = 11, *p* = 0.78), the mean value for a given condition was computed by taking the average between the two measures.

### 2.7. Statistical and psychometric analyses

The decision to use parametric or non-parametric statistical methods on a given data sample was motivated by the D'Agostino and Pearson omnibus normality test, which we performed on all analysed samples using GraphPad Prism software. First, the 75% just noticeable difference (JND) was computed for each individual participant by fitting a logistic psychometric function to the proportion of correct responses related to the increasing values of the amplitude of vibration and the relative reduction of TF. The mean psychometric curve was then computed based on the median values of the thresholds and slopes across all individual psychometric functions. The psychometric fitting and model comparison (5000 iterations) were performed using the dedicated functions of the PALAMEDES toolbox based on maximum-likelihood method [44].

## 3. Results

### 3.1. Frictional properties of the materials

The three interfacial materials were selected for their specific properties: aluminium (smooth and hydrophilic), polypropylene (smooth and hydrophobic) and polyurethane (stickier and hydrophobic). A first analysis was performed to quantify their frictional differences. The coefficient of dynamic friction (CF) of the materials was computed for all participants and showed a significant effect of the material (one-way repeated measures ANOVA with Geisser–Greenhouse correction: *F*_1.78,19.6_ = 22.82, *p* < 0.0001). The CF was similar for aluminium (Al) and polypropylene (PP) (Paired *t*-test: *N* = 12, d.f. = 11, *p* = 0.28) and significantly higher for polyurethane (PU) compared to both Al (paired *t*-test: *N* = 12, d.f. = 11, *p* = 0.0005) and PP (paired *t*-test: *N* = 12, d.f. = 11, *p* < 0.0001) (figure 1*d*). Thus, Al and PP were found to have very similar frictional properties whereas PU was significantly stickier. Since it has also been shown that the CF can increase between two consecutive slides [18], we compared it for the two consecutive slides composing each trial. No difference in CF was found between the first and second slide for any of the three materials (*t*-test with Bonferroni correction with all *p* > 0.05).

We also measured the moisture level of the skin for each experimental condition. For the three materials, we examined the correlation between the CF and the mean humidity level of the skin. The Pearson correlation revealed a significant positive relationship between CF and fingertip humidity for Al (*n* = 12, *R* = 0.72, *p* = 0.009) and PP (*n* = 12, *R* = 0.77, *p* = 0.004) as well as an almost significant positive trend for PU (*n* = 12, *R* = 0.54, *p* = 0.070). Thus, the CF was found to be greater for more humid fingers on all materials (figure 1*e*).

### 3.2. Human sensing of frictional changes: absolute or relative?

When the device was kept static against the finger during the ultrasonic stimulation, participant answers were around the chance level independently of the amplitude of ultrasonic vibration (figure 2*b*). Despite some extreme values for lower amplitudes, the higher amplitudes, which are more prone to possible artefacts generated by the power circuits, did not exhibit values suggesting a specific perception. The absence of psychometric trend during static stimulation showed that detection in the condition of dynamic touch was not due to a perceptual artefact produced by the experimental set-up. In order to provide a direct comparison with previous studies, we defined the psychophysical thresholds for detecting the reduction of tangential force (TF) by the 75% just noticeable difference (JND) between the slide containing the ultrasonic pulses and the one without ultrasonic stimulation. In the condition with lateral movement against the surface, the 75% JND was estimated for each individual participant and the mean psychometric curve for all participants was then computed out of the median of the individual slopes and psychophysical thresholds. The amplitudes of the ultrasonic vibrations at the 75% JND were 0.17 µm (IQR = 0.34–0.11), 0.23 µm (IQR = 0.30–0.20), and 0.27 µm (IQR = 0.36–0.21) for Al, PP and PU, respectively (figure 2*c*).

The data from the force sensor was then used to estimate the relationship between friction reduction and its perception. Considering Weber's Law of JND [45], we examined not only the net reduction of TF induced by the ultrasonic pulse at threshold, but also the percentage of reduction compared to the value of TF measured during the 100 ms pre-stimulus interval (relative reduction of TF). In the amplitude range of our study, the net reduction of TF (figure 2*d*) and its ratio to the pre-stimulus TF (figure 2*e*) were found to increase linearly with the amplitude of the ultrasonic vibration. The goodness of fit was assessed by the *R*^2^ coefficients, which were respectively 0.98 (Al), 0.99 (PP) and 0.98 (PU) for the net reduction of TF and 0.94 (Al), 0.99 (PP) and 0.98 (PU) for the relative reduction of TF. Since the normal force was kept constant, this measure reflected the relative decrease in CF. For any threshold amplitude of ultrasonic vibration, we could estimate the reduction of the TF by linear interpolation of the measures obtained for the pre-defined levels of stimulation enclosing the threshold.

**Figure 3.**
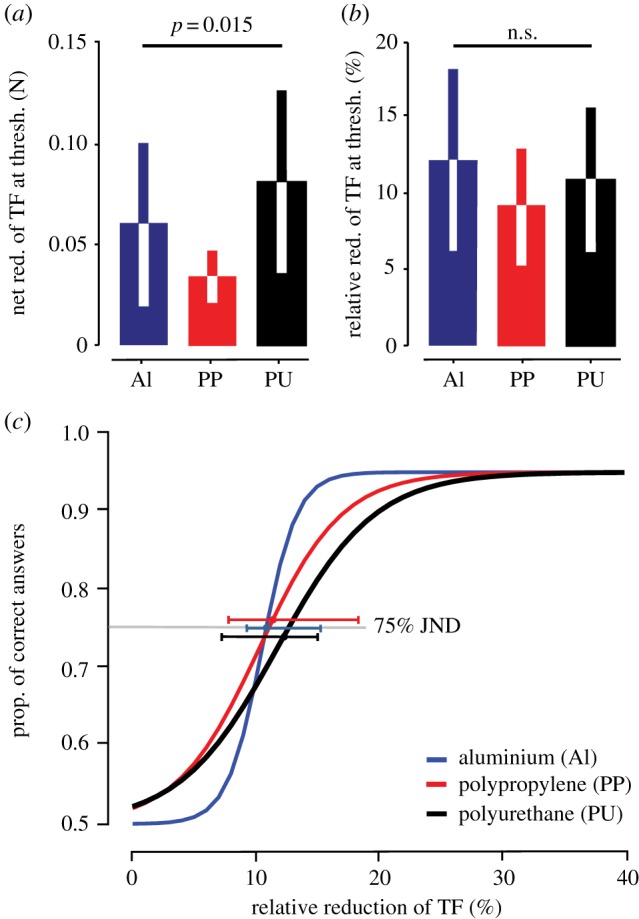
(*a*) Net reduction of friction computed at the individual psychophysical thresholds, for the three tested materials (mean ± s.d.). (*b*) Relative reduction of TF computed at the individual psychophysical threshold (mean ± s.d.). (*c*) The psychometric function computed out of the median of the individual slopes and psychophysical thresholds (median ± IQR) was computed for each material (the three curves are slightly shifted for better visibility: Al is accurate, PP is shifted by 0.01 upwards and PU is shifted by 0.01 downwards).

The net reduction of TF at the 75% JND was, on average, quite different across the three materials: 0.060 ± 0.042 N, 0.034 ± 0.011 N and 0.083 ± 0.045 N for Al, PP and PU, respectively (figure 3*a*). A one-way repeated-measures ANOVA with Geisser–Greenhouse correction confirmed that the net reduction of TF at threshold was significantly different across the three materials (*F*_1.556,17.11_ = 5.97, *p* = 0.015). In contrast, when the reduction of TF at threshold was expressed relative to the initial TF, very similar values were observed across the three materials (12.0 ± 5.6%, 9.0 ± 4.3% and 10.6 ± 5.0% for Al, PP and PU, respectively) (figure 3*b*) and the one-way repeated-measures ANOVA with Geisser–Greenhouse correction showed no significant difference across the three materials (*F*_1.427,15.7_ = 1.58, *p* = 0.23). The participant's psychophysical threshold was also found to be consistent across the three conditions for both the net reduction of TF (ANOVA's matching effectiveness: *F*_11,55_ = 1.97, *p* = 0.050) and relative reduction of TF, (ANOVA's matching effectiveness: *F*_11,22_ = 2.45, *p* = 0.036).

In a second analysis, the psychometric stimulus–response curves were also computed for each of the three materials directly from the measurements of the interfacial forces. For each participant, individual trials were distributed in 10 intervals depending on the measured reduction of TF. The 10th to 90th percentiles of TF reduction were defined as cut-off values in order to define the borders of these intervals. The intervals were different across participants and depended on their specific skin–surface friction. For all intervals where TF was reduced, performance was estimated as the proportion of correct answers and was fitted with a logistic psychometric function to compute the related psychometric curve. The mean psychometric curve for all participants was then computed out of the median of the individual slopes and psychophysical thresholds (figure 3*c*). The 75% JND were then compared. Very similar threshold values were observed across the three materials with 10.5% (IQR = 16.0–9.2), 10.2% (IQR = 18.7–7.4) and 11.8% (IQR = 15.0–6.9) for Al, PP and PU, respectively. A Friedman test showed no significant difference across the three materials (*χ*^2^ = 0.67, *p* = 0.72). Thus, both analyses suggest that friction perception follows Weber's Law.

### 3.3. Influence of the properties of the skin

**Figure 4.**
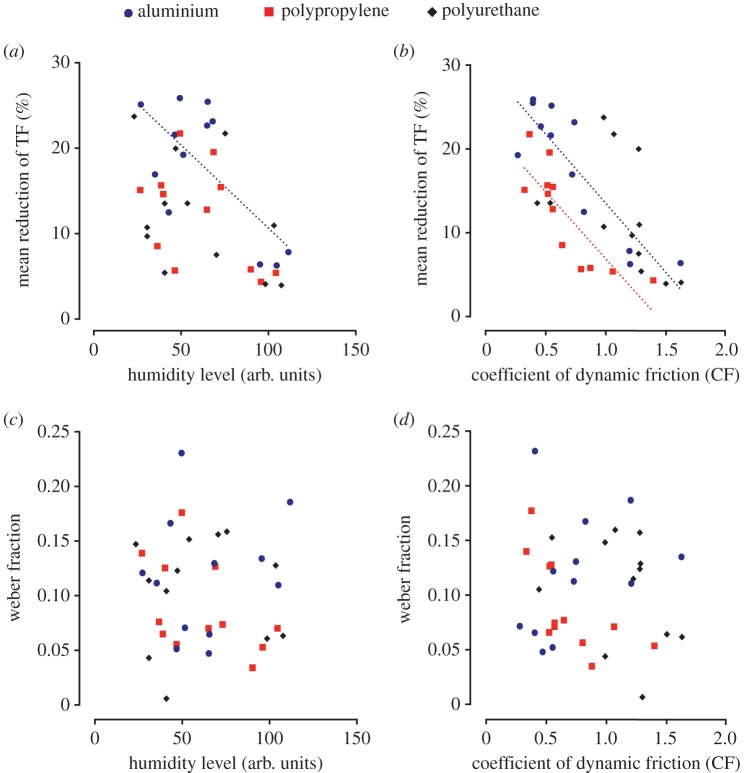
(*a*) Pearson correlation between the individual humidity level of the skin and the mean reduction of TF for each material. The correlation was significant for aluminium (dashed line). (*b*) Pearson correlation between the individual coefficient of dynamic friction and the mean reduction of TF. The correlation, represented on the figure by dashed lines, was strongly significant for aluminium and polypropylene. (*c*) Pearson correlation between the individual humidity level of the skin of the participants and the participants' Weber fractions. (*d*) Pearson correlation between the coefficient of dynamic friction and the participants' Weber fractions. (Online version in colour.)

We assessed the influence of fingertip moisture level and coefficient of dynamic friction on the mean relative reduction of TF achieved by the ultrasonic vibration as well as on the Weber fraction. The mean relative reduction of TF for each material was computed for each participant by averaging the reduction of TF across all the intensities of ultrasonic vibration. A Pearson's statistical analysis with Bonferroni correction showed a significant negative correlation (*n* = 12, *R* = −0.70, *p* = 0.036) between humidity level and mean relative reduction of TF only for aluminium (figure 4*a*). PP (*n* = 12, *R* = −0.48, *p* = 0.11) and PU (*n* = 12, *R* = −0.43, *p* = 0.16) exhibited similar but non-significant trends. On the other hand, the Bonferroni corrected correlation between the CF and the mean percentage of TF reduction (figure 4*b*) was found strongly significant for aluminium (*n* = 12, *R* = −0.88, *p* = 0.0006) and polypropylene (*n* = 12, *R* = -0.83, *p* = 0.0003) but not for PU (*n* = 12, *R* = −0.46, *p* = 0.13). These results suggest that, on smooth materials, ultrasonic vibrations generate less reduction of the tangential force when applied to stickier fingers. A Pearson analysis was also performed with the Weber fraction and did not show a significant correlation with the humidity level (figure 4*c*) for any material (Al: *n* = 12, *R* = 0.15, *p* = 0.65; PP: *n* = 12, *R* = −0.48, *p*= 0.11; PU: *n* = 12, *R* = 0.00, *p* = 0.99). The correlation was also not significant between the Weber fraction and the CF (figure 4*d*) for any material (Al: *n* = 12, *R* = 0.32, *p* = 0.32; PP: *n* = 12, *R* = −0.66, *p* = 0.06; PU: *n* = 12, *R* = −0.34, *p* = 0.27). Overall, the humidity and stickiness of the fingertip seemed to affect the amount of ultrasonic friction reduction but did not influence the individual Weber fraction for detecting changes in friction.

## 4. Discussion

Participants exhibited a very accurate sensitivity to transient modulations of friction induced by ultrasonic lubrication during a passive dynamic touch experiment, in which the speed of sliding and the normal force between the finger and the device were kept constant. The current study was performed with a new-generation ultrasonic tactile display controlled in closed loop in order to deliver the same amplitude of ultrasonic vibration to each participant independently of the mechanical properties of the fingertip [24]. Participants consistently detected very subtle changes in tangential force (TF) around 0.04–0.05 N, which corresponded to a reduction of 11%, i.e. a Weber fraction of 0.11, and their performance was consistent across the three conditions. The Weber fraction across touched materials with varying frictional properties remained constant, thus suggesting that detection was truly mediated by the friction reduction relative to its initial level. The results showed a positive correlation between the dynamic CF and the humidity level of the skin. Such an increase was already observed in previous studies on tactile exploration [20,46,47]. The relationship between CF and humidity can also display a bell-shaped profile with lower CF measured for dry and very wet fingers during grip [48] or when water is additionally added [49]. During grip or static touch, the sweat, whose production does not depend on the motion of the fingertip [2], is trapped in the contact area and can accumulate into an intermediate water layer that acts as a lubricant. During tactile exploration as performed in this study, the movement of the ultrasonic device against the finger enables the fingerpad to deposit some of its excess sweat on the surface [46], which prevents the formation of a lubricating water film at very high moisture levels. The humidity of the skin and its stickiness also played a role in the ultrasonic friction reduction by decreasing the magnitude of the relative reduction of TF achieved by the ultrasonic vibration. The correlation between the humidity of the fingertip and the relative reduction of TF was not observed in a recent study on ultrasonic lubrication [26], probably because of the very high stimulation amplitudes that were used (3 µm producing up to 95% reduction in CF), which could have overcome the influence of the molecular bonds formed at the interfacial water layer. However, our finding is in line with recent results showing larger frictional differences between materials for dry fingers during tactile interaction on flat surfaces [20].

It has been shown in a recent study that skin stretches induced by friction are translated into strains spreading on the surface of the skin [1] and these strains can activate specific populations of mechanosensitive afferents [5,50–52]. A neuronal coding of the spatio-temporal properties of skin strains was also observed in the cuneate nucleus [53] and in the primary somatosensory cortex [54]. However, the mechanisms underlying the integration of these frictional cues and their cognitive representation are largely unknown. It is only the recent development of novel devices able to modulate selectively friction in a controlled fashion that have enabled a systematic investigation of friction perception. Ultrasonic or electrostatic friction modulation has already been used to investigate the human perception of friction [36,37,55] but the current study is the first to investigate the parameters mediating and influencing perception of transient changes of friction. The observed Weber fraction obtained in controlled passive touch conditions with our experimental paradigm was 0.11, which shows that the sensitivity to transient changes in friction is higher than the sensitivity to different frictional levels between distinct surfaces [36,37]. In [36], the two compared surfaces had different frictional levels but the friction was kept uniform within the surfaces. This experimental procedure is more demanding than detecting sudden transitions as it requires maintaining a representation of the friction generated by the preceding exploration in working memory and, hence, it is not surprising that the reported Weber fraction was higher than in the present study. Furthermore, only one participant was tested and it is impossible to determine how sensitive to changes in friction that participant was compared to the general population. In [37], the frictional levels to be compared were located on the same interface but the psychophysical procedure was not a forced-choice task. Instead, participants were asked if they felt a difference of friction between the two sides of the plate with the possibility to say no. This method often induces the participants to be more conservative about reporting a sensation. It is also possible that the sharpness of the transition between high and low friction could influence the psychophysical threshold. Indeed, a recent study showed that a 2 ms variation in the rise time of an ultrasonic signal was consistently detected by humans [56]. In our study, the ultrasonic signal of our device was very close to a perfect square with only 1.5 ms needed to achieve the desired level of ultrasonic vibration. Hence it probably generated particularly salient frictional steps. This salience was further increased by the fact that the stimuli consisted of two rapidly succeeding pulses. The Weber fraction is also lower in comparison to most perceptual cues. For example, the Weber fractions for differential discrimination reported in literature are 0.34 for viscosity [57], 0.43 for thermal diffusivity on glabrous skin [58], 0.23 for stiffness [59] and 0.07 for grasping force [60]. These results show that touch is particularly efficient at detecting the application of forces on the fingertip.

The results of our study confirm the high tactile sensitivity of the human fingertip to frictional cues and supports the validity of Weber's Law for the JND of frictional changes. This outcome is important considering that Weber's Law is not necessarily valid for all types of stimuli including in the tactile sense when we discriminate the intensities of vibrotactile signals [33] or during the tactile discrimination of length through the finger-span method [35]. The activity of fast adapting type I afferents has also specifically been shown to be less sensitive to differences in intensity when the intensity of the reference vibration is low [61] while these afferents are known to be particularly sensitive to transient changes of friction [62]. In [60], an intensity discrimination psychophysical task was performed on a large range of vibrational intensities. The participant's sensitivity was assessed with a forced-choice two-interval procedure and a Zwilocki staircase, which estimated the 75% JND. The Weber fraction was shown to be significantly higher for intensities of the reference stimulus lower than 7 dB. Using an identical psychophysical tracking and a two-interval forced-choice task, Gescheider *et al.* [33] reported a violation of Weber's Law for the human perception of sinusoidal vibrations or narrow-band noise applied on the thenar eminence. In this study, the stimulus consisted of a short increment of a baseline level of vibration that was randomly located in one of two consecutive time intervals and participants were asked to report the correct interval. A large deviation from Weber's Law was observed for very low levels of baseline vibration. Therefore, the perception of transient changes in friction might similarly show deviations from Weber's Law at very low levels of friction. In our study, stickiness ranged from relatively low (aluminium and polypropylene) to moderately high for polyurethane. In future work, it could be possible to test even lower baseline frictions by using a baseline level of ultrasonic vibration to reduce the pre-stimulation finger-surface friction, which would be further reduced by short increments of the ultrasonic vibration.

The study has also direct implications for the nascent industry of tactile displays with friction mediated tactile feedback. Our results suggest that the simulation of tactile features by suddenly decreasing or increasing the friction should take into account the pre-stimulation frictional level in order to achieve a repeatable perception throughout prolonged interaction. More generally, the Weber fraction related to a stepwise decreasing and increasing of friction is an essential information for manufacturers in order to provide displays that modulate friction with amplitude ranges relevant to the observed psychophysical thresholds in the population.

## Data accessibility

The data analysis was performed using an acquired dataset of 2.63 Gb and several in-house Matlab scripts that will be made publicly available in a Dryad repository.
